# Apparent Insulin Deficiency in an Adult African Population With New-Onset Type 2 Diabetes

**DOI:** 10.3389/fcdhc.2022.944483

**Published:** 2022-07-28

**Authors:** Davis Kibirige, Isaac Sekitoleko, Priscilla Balungi, William Lumu, Moffat J. Nyirenda

**Affiliations:** ^1^ Non-Communicable Diseases Program, Medical Research Council/Uganda Virus Research Institute and London School of Hygiene and Tropical Medicine Uganda Research Unit, Entebbe, Uganda; ^2^ Department of Non-Communicable Diseases Epidemiology, Faculty of Epidemiology and Population Health, London School of Hygiene and Tropical Medicine, London, United Kingdom; ^3^ Clinical Diagnostics Laboratory Services, Medical Research Council/Uganda Virus Research Institute and London School of Hygiene and Tropical Medicine Uganda Research Unit, Entebbe, Uganda; ^4^ Department of Medicine, Mengo Hospital, Kampala, Uganda

**Keywords:** insulin deficiency, adult, newly diabetes mellitus, Uganda, Sub-Sahara Africa

## Abstract

Identifying patients with new-onset type 2 diabetes who have insulin deficiency can aid in timely insulin replacement therapy. In this study, we measured fasting C-peptide concentration to assess endogenous insulin secretion and determine the prevalence and characteristics of patients with insulin deficiency in adult Ugandan patients with confirmed type 2 diabetes at presentation.

**Methods:**

Adult patients with new-onset diabetes were recruited from seven tertiary hospitals in Uganda. Participants who were positive for the three islet autoantibodies were excluded. Fasting C-peptide concentrations were measured in 494 adult patients, and insulin deficiency was defined as a fasting C-peptide concentration <0.76 ng/ml. The socio-demographic, clinical, and metabolic characteristics of participants with and without insulin deficiency were compared. Multivariate analysis was performed to identify independent predictors of insulin deficiency.

**Results:**

The median (IQR) age, glycated haemoglobin (HbA1c), and fasting C-peptide of the participants was 48 (39-58) years,10.4 (7.7-12.5) % or 90 (61-113) mmol/mol, and 1.4 (0.8-2.1) ng/ml, respectively. Insulin deficiency was present in 108 (21.9%) participants. Participants with confirmed insulin deficiency were more likely to be male (53.7% *vs* 40.4%, p=0.01), and had a lower body mass index or BMI [p<0.001], were less likely to be hypertensive [p=0.03], had reduced levels of triglycerides, uric acid, and leptin concentrations [p<0.001]), but higher HbA1c concentration (p=0.004). On multivariate analysis, BMI (AOR 0.89, 95% CI 0.85-0.94, p<0.001), non-HDLC (AOR 0.77, 95% CI 0.61-0.97, p=0.026), and HbA1c concentrations (AOR 1.08, 95% CI 1.00-1.17, p=0.049) were independent predictors of insulin deficiency.

**Conclusion:**

Insulin deficiency was prevalent in this population, occurring in about 1 in every 5 patients. Participants with insulin deficiency were more likely to have high HbA1c and fewer markers of adiposity and metabolic syndrome. These features should increase suspicion of insulin deficiency and guide targeted testing and insulin replacement therapy.

## Introduction

Measurement of fasting, random, or stimulated C-peptide concentrations, as an indicator of pancreatic beta-cell insulin secretion, is recommended in patients with new-onset diabetes to identify specific diabetes subtypes (type 1 and type 2 diabetes and maturity-onset diabetes of the young), patients that require timely insulin replacement therapy, and also to predict response to oral hypoglycaemic agents (1, 2). In patients with type 1 or type 2 diabetes and absolute insulin deficiency, early initiation of insulin therapy at diagnosis helps in achieving early optimal glycaemic control, preserves and improves beta-cell mass and function, in addition to averting diabetic ketoacidosis, and early onset of diabetes complications (3).

While fasting or random C-peptide concentrations are commonly measured to guide the management of diabetes in high-income countries, it remains a less available and expensive test in resource-constrained settings like sub-Saharan Africa (SSA). Initiation of insulin or switching from oral hypoglycaemic agents to insulin therapy in adult patients with new-onset or long-standing diabetes and presumed insulin deficiency in such settings is based on specific clinical suspicion. This has potential limitations, in part because there is little data on the prevalence, characteristics, and clinical correlates of insulin deficiency in adult patients with new-onset type 2 diabetes in SSA.

To address this evidence gap, we undertook this sub-study which was part of the Uganda Diabetes Phenotype (UDIP) study that aimed to investigate the manifestation of diabetes in adult Ugandan patients with recently diagnosed diabetes. In this sub-study, we specifically sought to determine the prevalence, characterisation, and predictors of insulin deficiency in an adult population with newly diagnosed type 2 diabetes in Uganda.

## Materials and Methods

### Study Setting, Duration, and Participants

The UDIP study was carried out in seven tertiary public and private hospitals located in Central and Southwestern Uganda between February 2019 and October 2020. These hospitals predominantly serve the surrounding urban, peri-urban, and rural populations and have once weekly outpatient diabetes clinics for long-term management of adult patients with diabetes.

A total of 494 participants were recruited from these outpatient diabetes clinics. The inclusion criteria were patients aged ≥18 years with a recent diagnosis of diabetes (<3 months since diagnosis), initiated on any glucose-lowering treatment or treatment naïve, and tested negative for the three measured islet autoantibodies (defined as a concentration of autoantibodies against glutamic acid decarboxylase-65 [GADA], zinc transporter 8 [ZnT8-A], and tyrosine phosphatase [IA-2A] of ≤34U/ml, ≤67.7 U/ml, and ≤58 U/ml, respectively).

Participants who were critically ill and required urgent hospital admission were not immediately recruited into the study. Enrolment into the study was done later, at least two weeks after discharge from the hospital (but within three months of diagnosis), when they re-attended the outpatient diabetes clinic in a more clinically stable state. Pregnant women with new-onset diabetes were excluded from the study.

### Assessment of Socio-Demographic, Clinical, and Biophysical Characteristics

Participants were recruited in the study after an overnight fast of a minimum of eight hours. Using pre-tested case report forms, we collected information on relevant socio-demographic and clinical characteristics (age at diagnosis, gender, residence, smoking and alcohol ingestion habits, history of admission at the time of diagnosis, presence of urine or serum ketosis on admission, co-existing medical conditions, and glucose-lowering diabetes therapies initiated).

This was followed by resting blood pressure and anthropometric measurements (weight, height, waist circumference or WC, hip circumference or HC, body mass index or BMI, waist: hip circumference ratio or WHR, and waist circumference: height ratio or WHtR).

### Assessment of Metabolic Characteristics and Laboratory Measurements

Following standardised procedures, a fasting venous blood sample was drawn for measurement of blood glucose (FBG), glycated haemoglobin (HbA1c), insulin, C-peptide, lipid profile, uric acid, leptin, and three islet autoantibodies (GADA, ZnT8-A, and IA-2A). This was followed by a 75-gram oral glucose tolerance test (OGTT), with venous blood samples being drawn again 30 and 120 minutes following glucose solution ingestion to determine the serum glucose, insulin, and C-peptide concentrations at those two time-points.

All the above tests were performed at the ISO-certified clinical chemistry laboratory at the Medical Research Council/Uganda Virus Research Institute and London School of Hygiene and Tropical Medicine Uganda Research Unit, Entebbe Uganda using electro-chemiluminescence immunoassays manufactured by Roche diagnostics Limited, Germany on a Cobas 6000 C-model SN 14H3-15 machine (Hitachi High Technologies Corporation, Tokyo Japan). Pancreatic autoantibody testing was done using autoantibody ELISA kits from RSR Limited (Cardiff CF14 5DU, UK).

To determine the insulin resistance (homeostatic model assessment-2 [HOMA2] insulin resistance or HOMA2-IR) and the pancreatic beta-cell function (HOMA2-%B), we used the online HOMA2 calculator by the Diabetes Trial Unit of the University of Oxford, Oxford UK (4). Pancreatic beta-cell function was also assessed using an optimal marker of beta-cell function- the oral insulinogenic index (IGI) using the formula: IGI = δ insulin (30 min insulin - 0 min insulin in µU/ml)/δ glucose (30 min glucose - 0 min glucose in mmol/l) (5). We used the online quantitative insulin sensitivity check index (QUICKI) calculator to calculate the QUICKI using the fasting serum glucose and insulin concentrations (6).

### Definition of Insulin Deficiency

Insulin deficiency was defined as a fasting C-peptide concentration <0.76 ng/ml (equivalent to 0.25 nmol/l or 250 pmol/l). Absolute insulin deficiency or requirement was defined as a fasting C-peptide concentration <0.24 ng/ml (equivalent to 0.08 nmol/l or 80 pmol/l) (1, 2).

### Statistical Analysis

The categorical and continuous variables were expressed as proportions and medians with inter-quartile range (IQR), respectively. The prevalence of insulin deficiency was expressed as a frequency. Differences in the socio-demographic, clinical, anthropometric, and metabolic characteristics of participants with and without insulin deficiency were analysed using *the x* ^2^ test for categorical data and the Kruskal Wallis test for continuous data. Multivariate analysis was performed to identify predictors of insulin deficiency. All analyses were done using STATA statistical software version 15 College Station, TX: StataCorp LLC.

### Ethical Approval

This study was approved by the Research Ethics Committee of Uganda Virus Research Centre, Entebbe Uganda (GC/127/18/05/650) and the Uganda National Council of Science and Technology (HS 2431). Administrative clearance was also obtained from all participating study sites. All enrolled study participants provided written informed consent to participate in the study.

## Results

### Baseline Characteristics of All Study Participants

The socio-demographic, clinical, anthropometric, and metabolic characteristics of all study participants are summarised in **Table 1**.

**Table 1:** Socio-demographic, clinical, anthropometric, and metabolic characteristics of the study participants with and without insulin deficiency.

Characteristic	All study participants (n=494)	Insulin deficient, (n=108, 21.9%)	Insulin sufficient, (n=386, 78.1%)	P value
**Socio-demographic and clinical**				
Age, years	48 (39-58)	48 (39-58)	48 (39-57)	1.00
Gender Male	214 (43.3)	58 (53.7)	156 (40.4)	0.01
Female	280 (56.7)	50 (46.3)	230 (59.6)	
Residence Urban Rural	364 (73.8) 127 (26.2)	79 (73.2) 29 (26.8)	285 (74.0) 98 (26.0)	0.73
Current alcohol ingestion	112 (22.9)	21 (20.0)	91 (23.6)	0.69
Past and current history of smoking	39 (7.9)	13 (2.6)	26 (5.3)	0.16
History of admission at diagnosis	199 (40.5)	55 (50.9)	144 (37.5)	0.02
Presence of urine or serum ketosis on admission	69 (30.7)	27 (45.8)	42 (25.3)	0.03
Glucose-lowering drugs used**				
Diet alone	18 (3.6)	3 (2.8)	15 (3.9)	0.78
Metformin	398 (80.6)	71 (65.7)	327 (84.7)	<0.001
SU	189 (38.3)	34 (31.5)	155 (40.2)	0.10
Insulin	132 (26.7)	48 (44.4)	84 (21.8)	<0.001
Medical comorbidities				
Hypertension	171 (34.6)	28 (25.9)	143 (37.1)	0.03
HIV infection	58 (11.7)	10 (9.3)	48 (12.4)	0.37
Tuberculosis	2 (0.4)	1 (0.3)	1 (0.9)	0.39
Systolic BP, mmHg	126 (115-137)	122 (111-131)	126 (117-140)	0.05
Diastolic BP, mmHg	84 (77-91)	81 (75-86)	85 (77-92)	0.02
**Anthropometry**				
BMI, kg/m^2^	27.5 (23.5-31.5)	24.3 (21.0-28.2)	28.1 (24.6-32.1)	<0.001
BMI in kg/m^2^ < 25	161 (32.9)	57 (52.8)	104 (27.2)	<0.001
WC, cm	96.0 (87.0-104.5)	88.9 (81.5-96.5)	98.5 (91.0-106.4)	<0.001
HC, cm	103.0 (96.0-111.5)	98.0 (90.1-106.5)	104.0 (96.5-113.0)	<0.001
WHR	0.92 (0.88-0.96)	0.91 (0.87-0.95)	0.93 (0.88-0.97)	0.06
WC: height ratio	0.59 (0.53-0.65)	0.54 (0.50-0.59)	0.61 (0.55-0.66)	<0.001
**Metabolic**				
TC, mmol/l	4.0 (3.3-5.0)	3.7 (3.1-4.6)	4.1 (3.3-5.1)	0.03
HDLC, mmol/l	1.0 (0.7-1.2)	0.9 (0.7-1.2)	1.0 (0.8-1.2)	0.68
TGL, mmol/l	1.3 (1.0-1.8)	1.2 (0.9-1.7)	1.4 (1.0-1.9)	0.02
LDLC, mmol/l	2.6 (1.9-3.4)	2.4 (1.7-3.0)	2.7 (2.0-3.5)	0.14
Non-HDLC, mmol/l	3.0 (2.4-3.8)	2.7 (2.1-3.6)	3.1 (2.5-3.9)	0.01
Uric acid, µmol/l	273.0 (222.0-335.0)	240.5 (197.5-294.5)	284.0 (232.0-342.0)	<0.001
Leptin, pmol/l	2538.3 (619.8-5495.5)	858.0 (279.0-2834.5)	3207.5 (904.5-6276.0)	<0.001
HbA1c, %	10.4 (7.7-12.5)	11.5 (8.9-13.4)	10.0 (7.4-12.2)	0.004
HbA1c, mmol/mol	90 (61-113)	102 (75-123)	86 (57-110)	0.004
Fasting glucose, mmol/l	8.5 (6.2-13.4)	10.4 (7.0-15.2)	8.1 (6.0-12.7)	0.001
Fasting serum insulin, µU/ml	5.9 (3.0-10.6)	2.2 (0.9-3.9)	7.3 (4.5-12.2)	<0.001
Fasting C-peptide, ng/ml	1.4 (0.8-2.1)	0.5 (0.2-0.6)	1.7 (1.3-2.4)	<0.001
30-minute glucose, mmol/l	13.0 (9.9-18.3)	15.9 (11.3-20.8)	12.4 (9.7-16.9)	<0.001
30-minute insulin, µU/ml	11.1 (5.5-22.5)	3.7 (1.7-7.4)	14.4 (7.7-25.3)	<0.001
30-minute C-peptide, ng/ml	2.1 (1.1-3.3)	0.7 (0.4-1.1)	2.5 (1.7-3.8)	<0.001
120-minute glucose, mmol/l	17.2 (12.4-23.4)	20.9 (16.0-28.1)	16.2 (11.6-22.4)	<0.001
120-minute insulin, µU/ml	13.8 (6.9-27.5)	5.7 (2.1-11.0)	17.2 (8.8-33.5)	<0.001
120-minute C-peptide, ng/ml	2.8 (1.5-4.8)	1.11 (0.5-1.8)	3.3 (2.1-5.3)	<0.001
HOMA2-IR	1.2 (0.8-2.0)	0.8 (0.6-1.3)	1.3 (0.8-2.1)	0.01
QUICKI	0.35 (0.31-0.42)	0.41 (0.36-0.52)	0.34 (0.31-0.39)	<0.001
HOMA2-%B	43.1 (20.7-77.6)	15.4 (9.2-36.1)	46.1 (22.5-79.8)	0.007
Oral IGI	1.3 (0.5-3.9)	0.5 (0.2-1.0)	1.8 (0.6-4.6)	<0.001
TNF-α	24.5 (19.5-34.0)	22.0 (17.8-35.0)	25.2 (20.0-34.0)	0.06
IL-1β	16.0 (14.0-20.2)	15.8 (13.8-20.0)	16.0 (14.0-20.3)	1.00
IL-6	23.0 (15.0-43.5)	24.0 (15.0-44.5)	23.0 (15.0-40.3)	0.78

** Used as monotherapy or in combination. Data is presented in form of percentages for categorical variables and median with IQR for continuous variables.

SU, Sulfonylureas.

The median (IQR) age, BMI, HbA1c, and fasting C-peptide of the participants was 48 (39-58) years, 27.5 (23.5-31.5) kg/m^2^, 10.4 (7.7-12.5) % or 90 (61-113) mmol/mol, and 1.4 (0.8-2.1) ng/ml, respectively, with 56.7% of participants being female. About 41% of participants reported a history of admission at the time of diagnosis with 30.7% of those admitted presenting with urine or serum ketosis. The majority of the participants were initiated on metformin, either as monotherapy or in combination with other therapies (80.6%), with only 26.7% initiated on insulin therapy.

### Prevalence of Insulin Deficiency

One hundred and eight participants had insulin deficiency, corresponding to a prevalence of 21.9%. Absolute insulin deficiency or requirement was noted in 26 (5.3%) participants.

### Socio-Demographic, Clinical, Anthropometric, and Metabolic Characterisation of Participants With and Without Insulin Deficiency

The socio-demographic, clinical, anthropometric, and metabolic characteristics of participants with and without insulin deficiency are summarised in **Table 1**.

Compared with those who were insulin sufficient, participants with confirmed insulin deficiency were more likely to be male (53.7% vs 40.4%, p=0.01), to be admitted at the time of diagnosis (50.9% vs 37.5%, p=0.02), to present with urine or serum ketosis on admission (45.8% vs 25.3%, p=0.03), and to be initiated on insulin at diagnosis (44.4% vs 21.8%, p<0.001). No statistically significant differences were noted in the age at diagnosis between the two groups.

Participants with insulin deficiency had statistically significant higher glycaemic indices (FBG-10.4 [7.0-15.2] vs 8.1 [6.0-12.7] mmol/l, p=0.001, HbA1c- 11.5 [8.9-13.4] vs 10 [7.4-12.2] % or 102 [75-123] vs 86 [57-110] mmol/mol, p=0.004 and 30-minute glucose- 15.9 [11.3-20.8] vs 12.4 [9.7-16.9] mmol/l, p<0.001) and a lower HOMA2-IR (0.8 [0.6-1.3] vs 1.3 [0.8-2.1], p=0.01). In addition, these participants had statistically significant lower markers of pancreatic beta cell function like HOMA2-B%, oral IGI, 30-and 120-minute C-peptide concentrations (p<0.001).

Compared with those who were insulin sufficient, participants with insulin deficiency were less likely to have hypertension comorbidity (25.9% vs 37.1%, p=0.03) and had statistically significant lower median BMI (24.3 [21.0-28.2] vs 28.1 [24.6-32.1] kg/m^2^, p<0.001), WC (88.9 [81.5-96.5] vs 98.5 [91.0-106.4] cm, p<0.001), triglyceride (1.2 [0.9-1.7] vs 1.4 [1.0-1.9] mmol/l, p=0.02), non-high-density lipoprotein cholesterol or non-HDLC (2.7 [2.1-3.6] vs 3.1 [2.5-3.9] mmol/l, p=0.01), uric acid (240.5 [197.5-294.5] vs 284 [232-342] µmol/l, p<0.001), and leptin (858 [279-2834.5] vs 3207.5 [904.5-6276], p=0.001) concentrations.

### Clinical Predictors of Insulin Deficiency

**Table 2** shows the independent predictors of insulin deficiency. On multivariate analysis, BMI (AOR 0.89, 95% CI 0.85-0.94, p<0.001), non-HDLC concentration (AOR 0.77, 95% CI 0.61-0.97, p=0.026), and HbA1c level (AOR 1.08, 95% CI 1.00-1.17, p=0.049) were noted to independently predict insulin deficiency in this study population.

**Table 2:** Multivariate analysis to identify the independent predictors of insulin deficiency.

Characteristic	AOR (95% CI)	P-value
Age at diagnosis, years	0.99 (0.97-1.01)	0.53
Male gender	1.30 (0.77-2.20)	0.32
Body mass index, kg/m^2^	0.89 (0.85-0.94)	<0.001
Non-HDLC concentration	0.77 (0.61-0.97)	0.03
HbA1c, %	1.08 (1.00-1.17)	0.05

AOR, Adjusted odds ratio; CI, confidence intervals; HbA1c, Glycated haemoglobin; HDLC, High-density lipoprotein cholesterol.

## Discussion

To our knowledge, this is the first study to robustly investigate the frequency, characterisation, and predictors of insulin deficiency in an adult African population with newly diagnosed confirmed type 2 diabetes. We demonstrate that the prevalence of insulin deficiency is relatively high, occurring in about 1 in every 5 adult patients with newly diagnosed type 2 diabetes. Absolute insulin deficiency or requirement, similar to what is described in most patients with type 1 diabetes, was also prevalent in our study population. The documented high prevalence of insulin deficiency in these participants with newly diagnosed type 2 diabetes is less likely to be due to pancreatic beta-cell exhaustion related to the natural disease progression, seen in patients with long-standing diabetes.

Studies that have evaluated pancreatic beta-cell function using fasting or random C-peptide measurement in adult populations with newly diagnosed type 2 diabetes in SSA are limited. The few available studies have been conducted in adult patients with long-standing type 2 diabetes, reporting a wide variation in the documented prevalence of insulin deficiency ranging from 7% to 73% (7–11). Except for one study which used a lower cut-off of fasting C-peptide concentration of <0.2 nmol/l (0.6 ng/ml) to define low C-peptide levels (7), the rest of the above studies used a cut-off of <0.3 nmol/l (0.9 ng/ml).

Varying degrees of insulin deficiency have been reported in Asian and Caucasian adult populations with new-onset type 2 diabetes, further underscoring phenotypic differences across ethnicities. Despite using varied parameters to define insulin deficiency (fasting and stimulated C-peptide, HOMA2-B%, and insulin sensitivity or HOMA2-S) and fasting C-peptide thresholds, a lower prevalence of insulin deficiency was reported in an adult South Korean (4.4%) (12) and Danish population (9.6%) (13) with new-onset presumed type 2 diabetes. Similar to our study findings, the Swedish All New Diabetics in Scania (ANDIS) cohort (14) and the India-Scotland Partnership for precision medicine in diabetes (INSPIRED) Indian cohort (15) also reported a relatively high frequency of insulin deficiency of 17.5% and 26.2%, respectively. Only the study performed on 500 adult patients with new-onset diabetes in Yemen reported a higher prevalence of insulin deficiency of 43.6%, based on HOMA2-B% and S (16).

In this study, participants who were insulin-deficient were more likely to be of the male gender, with a history of being admitted with ketosis and initiated on insulin therapy at the time of diagnosis of diabetes. A higher prevalence of insulin deficiency in male patients with type 2 diabetes has also been reported in two large studies conducted in Indian populations with long-standing type 2 diabetes (15, 17). Similar to what we observed in our study, male participants had a lower mean BMI compared to their female counterparts in one study that investigated novel diabetes subgroups in Indians with young-onset diabetes (17). The reasons to explain this clinical observation are not well-known and ought to be investigated.

In this study, we also demonstrated that insulin deficiency is associated with high glycaemic levels and reduced markers of adiposity and metabolic syndrome. An increase in BMI reduced the odds of insulin deficiency by 11%. A similar direct relationship between fasting C-peptide, as a measure of pancreatic beta-cell secretory function, and BMI has been reported in similar studies investigating pancreatic beta-cell function in African and Caucasian patients with long-standing and recently diagnosed diabetes (7–9, 18, 19). In addition to its role in regulating glucose homeostasis, insulin is an anabolic hormone that promotes lipogenesis and protein synthesis through the tyrosine kinase receptor pathway (20). Insulin deficiency, therefore, would result in an increased lipolysis, diffuse muscle wasting, and a low BMI phenotype as clinical hallmarks.

The association between an increase in HbA1c concentrations and insulin deficiency as shown in our study has been replicated in most studies, further highlighting the key role of insulin in glucose homeostasis (8, 9, 19, 21).

To our knowledge, non-HDLC concentration, as an indicator of increased cardiovascular disease risk, has not been reported in any study as a predictor of reduced pancreatic beta-cell secretory dysfunction. Conversely, an increase in non-HDLC concentrations reduced the odds of insulin deficiency in our study population. Most studies have reported a close relationship between increased TC, LDLC, TGL, and low HDLC concentrations and reduced insulin secretion by the pancreatic beta-cells (19, 22–24). This variation in study findings may be related to differences in diabetes phenotypes across populations. Participants with confirmed insulin deficiency in our study had a favourable lipid profile pattern (lower TC, LDLC, TGL, and non-HDLC concentrations).

Disorders in lipid metabolism have been implicated in pancreatic beta-cell dysfunction through their adverse effects of inhibiting glucokinase activity, glucose-stimulated insulin secretion, pancreatic beta-cell proliferation, in addition to inducing beta-cell apoptosis, and suppressing transcription of the pre-proinsulin gene (25, 26).

The reasons explaining the high frequency of insulin deficiency in our study population are unknown but may be related to structural pancreatic defects (presence of a naturally existing small beta-cell mass), local environmental (*in-utero* and early childhood chronic infections and malnutrition), and genetic factors that influence the capacity for beta-cell expansion (replication or neogenesis) or secretory function (27–30). The developmental origins of health and disease (DOHaD) concept emphasises the direct influence of chronic infections and undernutrition on structural development and physiologic function of most critical body organs during the early stages of life and development, hence increasing the future risk of metabolic conditions like type 2 diabetes (31). Undernutrition and chronic infections remain common in SSA and could explain the high rates of pancreatic beta-cell dysfunction and insulin deficiency noted in adult African patients with type 2 diabetes (32–36).

The African continent also has diverse populations with marked genetic heterogeneity. Black Africans may have susceptibility genes that directly affect pancreatic beta-cell mass and secretory function hence increasing the risk of developing type 2 diabetes. Two significant susceptibility genes that have been discovered to be highly prevalent in Western and Eastern African patients with type 2 diabetes and are worth mentioning are the Zinc Finger RANBP2-Type Containing 3 (ZRANB3) gene, which increases apoptosis resulting in reduced pancreatic beta-cell mass (37) and the transcription factor 7-like 2 (*TCF7L2*) gene, known to affect pancreatic beta-cell secretory function (38).

Because our study population did not include participants positive for islet autoantibodies and participants with insulin deficiency had a favourable lipid profile pattern (low TC, TGL, LDLC, and non-HDLC concentrations) and low circulating pro-inflammatory cytokines (IL-6, TNF-α, and IL-1β), we hypothesise that the observed insulin deficiency may be related to a structural defect (inadequate functional pancreatic beta-cell mass) either due to prior early-life environmental insults or genetic influences affecting the pancreatic beta-cell growth or regeneration and insulin secretion, as opposed to increased pancreatic beta-cell apoptosis due to lipotoxicity, increased endoplasmic reticulum stress, or inflammation.

In the context of SSA, communicable diseases like tuberculosis and HIV infection have been implicated in causing acute and chronic pancreatitis, hence reduced pancreatic beta-cell mass and consequent dysglycaemia (39, 40). Cases of brittle diabetes due to tuberculosis of the pancreas have been reported in SSA, a region with one of the highest burden of TB globally (39). In our study, pre-existing HIV and tuberculosis infections were only reported in 11.7% and 0.4% of participants, respectively with no differences between those who were insulin deficient and sufficient.

### Strengths and Limitations of the Study

Our study had several strengths. It recruited a substantially large number of patients with incident adult-onset diabetes without markers of islet autoimmunity from seven tertiary hospitals. Recruiting newly diagnosed adult patients rules out the possibility that the insulin deficiency observed in this study population is due to pancreatic beta-cell mass exhaustion which is part of the natural progressive nature of type 2 diabetes. We performed additional metabolic tests to further evaluate pancreatic beta-cell function in this study population like 30-and 120-minute C-peptide measurement, oral insulinogenic index, and HOMA2-%B calculation. To further characterise insulin deficiency in our study population, we performed additional relevant metabolic and immunological tests like leptin, uric acid, and pro-inflammatory cytokines. Few studies have performed these tests as part of their metabolic and immunologic characterisation.

Despite these strengths, the study had some limitations. Participants were recruited only from tertiary hospitals, which may introduce a selection bias and limit the generalisability of the findings to the adult Ugandan population with diabetes. Despite this limitation, it is important to note that the majority of patients self-refer to these tertiary hospitals for long-term diabetes management. Being cross-sectional in design, the study offers no evidence of a temporal relationship between type 2 diabetes and insulin deficiency.

## Conclusions

The relatively high prevalence of insulin deficiency in this Ugandan population with recently diagnosed adult-onset type 2 diabetes has significant clinical practice and policy implications in Uganda. It underscores the need for targeted assessment of pancreatic beta-cell function at diagnosis of diabetes, especially in patients with high glycaemic levels and reduced features of adiposity and metabolic syndrome. This will be important in identifying patients that require immediate insulin replacement therapy.

## Data Availability Statement

The original contributions presented in the study are included in the article/**Supplementary Material**. Further inquiries can be directed to the corresponding author.

## Ethics Statement

The studies involving human participants were reviewed and approved by Uganda Virus Research Centre, Entebbe Uganda and the Uganda National Council of Science and Technology. The patients/participants provided their written informed consent to participate in this study.

## Author Contributions

DK oversaw the entire data collection process and wrote the initial draft of the manuscript. IS performed the statistical analysis and reviewed all the versions of the manuscript. WL contributed to the discussion and reviewed all the versions of the manuscript. MN supervised this work, collectively contributed to the research idea, and reviewed all the versions of the manuscript. All authors contributed to the article and approved the submitted version.

## Funding

This study was funded by the UK Medical Research Council (MRC) and the UK Department for International Development (DFID) under the MRC/DFID Concordat agreement for the Medical Research Council/Uganda Virus Research Institute and London School of Hygiene and Tropical Medicine Uganda Research Unit, Entebbe Uganda (Project Reference: MC_UP_1204/16), and the National Institute for Health Research (NIHR) (17/63/131).

## Conflict of Interest

The authors declare that the research was conducted in the absence of any commercial or financial relationships that could be construed as a potential conflict of interest.

## Publisher’s Note

All claims expressed in this article are solely those of the authors and do not necessarily represent those of their affiliated organizations, or those of the publisher, the editors and the reviewers. Any product that may be evaluated in this article, or claim that may be made by its manufacturer, is not guaranteed or endorsed by the publisher.
